# Foamy Macrophages from Tuberculous Patients' Granulomas Constitute a Nutrient-Rich Reservoir for *M. tuberculosis* Persistence

**DOI:** 10.1371/journal.ppat.1000204

**Published:** 2008-11-11

**Authors:** Pascale Peyron, Julien Vaubourgeix, Yannick Poquet, Florence Levillain, Catherine Botanch, Fabienne Bardou, Mamadou Daffé, Jean-François Emile, Bruno Marchou, Pere-Joan Cardona, Chantal de Chastellier, Frédéric Altare

**Affiliations:** 1 CNRS, IPBS (Institut de Pharmacologie et de Biologie Structurale), Département Mécanismes Moléculaires des Infections Mycobactériennes, Toulouse, France; 2 Université de Toulouse, UPS, IPBS, F-31077 Toulouse, France; 3 Department of Pathology, Hôpital Ambroise Paré, Boulogne, France; 4 Service des Maladies Infectieuses et tropicales, Hôpital Purpan, Toulouse, France; 5 Unitat de Tuberculosi Experimental, Department of Microbiology, Fundació Institut per a la Investigació en Ciències de la Salut Germans Trias i Pujol and Universitat Autonoma de Barcelona, Badalona, Spain; 6 Aix Marseille Université, Faculté des Sciences de Luminy, Centre d'Immunologie de Marseille-Luminy (CIML), Marseille, France; 7 Inserm U631, CIML, Marseille, France; 8 CNRS, UMR6102, CIML, Marseille, France; Johns Hopkins School of Medicine, United States of America

## Abstract

Tuberculosis (TB) is characterized by a tight interplay between *Mycobacterium tuberculosis* and host cells within granulomas. These cellular aggregates restrict bacterial spreading, but do not kill all the bacilli, which can persist for years. In-depth investigation of *M. tuberculosis* interactions with granuloma-specific cell populations are needed to gain insight into mycobacterial persistence, and to better understand the physiopathology of the disease. We have analyzed the formation of foamy macrophages (FMs), a granuloma-specific cell population characterized by its high lipid content, and studied their interaction with the tubercle bacillus. Within our in vitro human granuloma model, *M. tuberculosis* long chain fatty acids, namely oxygenated mycolic acids (MA), triggered the differentiation of human monocyte-derived macrophages into FMs. In these cells, mycobacteria no longer replicated and switched to a dormant non-replicative state. Electron microscopy observation of *M. tuberculosis*–infected FMs showed that the mycobacteria-containing phagosomes migrate towards host cell lipid bodies (LB), a process which culminates with the engulfment of the bacillus into the lipid droplets and with the accumulation of lipids within the microbe. Altogether, our results suggest that oxygenated mycolic acids from *M. tuberculosis* play a crucial role in the differentiation of macrophages into FMs. These cells might constitute a reservoir used by the tubercle bacillus for long-term persistence within its human host, and could provide a relevant model for the screening of new antimicrobials against non-replicating persistent mycobacteria.

## Author Summary

*Mycobacterium tuberculosis*, the causative agent of tuberculosis, is responsible for dramatic health problems globally. It is estimated that this pathogen infects one-third of the human population and causes three million deaths annually. Most individuals remain asymptomatic for several years before developing an active disease. In such individuals, the bacilli are not cleared but rather persist in a dormant state. Major goals of TB research are to (i) understand how the bacilli remain alive for years within infected individuals, and (ii) find how to prevent their reactivation and hence clinical disease. During dormancy, most of the bacilli are confined to granulomas that consist of well-defined aggregates of different host immune cells. Granulomas prevent spreading of bacilli. In this study, we analyzed the role of a particular cell population found within granulomas, the “foamy macrophages”. These cells are filled with droplets of lipids, a well-known nutrient for persistent bacilli. We found that within these cells, the bacilli do not replicate, but remain alive and seem to internalize host lipids. The foamy macrophages might thus constitute a reservoir for persisting bacilli within their human host, and could provide a relevant model for screening of new antimicrobials against non-replicating persistent mycobacteria.

## Introduction

Tuberculosis caused by *Mycobacterium tuberculosis* (*M.tb*) remains one of the leading causes of mortality in the world, with around 2 million deaths each year [1]. Most individuals remain asymptomatic after the primary infection with only 10% at risk of developing an active disease during their life [2]. In asymptomatic individuals, the bacilli are not cleared but rather persist in a dormant state, from which they may reactivate and induce clinical disease at later stages [3].

The prognosis of the disease depends on the host's efficiency to constrain the bacilli at the site of infection. When inhaled *M.tb* reach the lungs, they are internalized by lung macrophages. The latter trigger the accumulation at the infectious site of macrophages, lymphocytes and dendritic cells, to form a granuloma, which is a major histo-pathological feature of TB. Within granulomas, macrophages differentiate into epithelioïd cells (differentiated macrophages), and/or fuse to form multinucleated giant cells (MGC). Macrophages with large numbers of lipid-free vacuoles, as well as macrophages filled with lipid-containing bodies, also called foamy macrophages (FM) are also found within granulomatous structures in both experimental animal models and human disease [4],[5]. The above cells are surrounded by a rim of lymphocytes, and at later stages, a tight coat of fibroblasts encloses the structure [6]. Although the structure and cell composition of granulomas are well known, the biology of these inflammatory structures and, more specifically, the role of granuloma-specific cell types, remain largely unknown.

We have previously developed an in vitro model of human tuberculous granulomas to gain insight into the survival strategies of the tubercle bacillus within its human host. This model now enables the characterization of granuloma-specific cell types, and their modulation by *M.tb* [7]. The main advantage of this model over in vivo animal models or ex vivo human biopsy samples, is the availability of live granuloma cells which facilitates analysis of their cell biology. Using this model we have recently shown that, within granulomas, large multinucleated giant cells, also known as Langhans giant cells, result from the induction of granuloma macrophage fusion by *M.tb* glycolipids [8]. We have shown that these cells have lost the ability to mediate bacterial uptake upon maturation, but have conserved their ability to mediate antigen presentation [9].

The differentiation of macrophages into FMs has been particularly well described in individuals developing a postprimary, also known as secondary or adult, TB. These postprimary infections are considered to be the result of re-infection or reactivation of a primary TB [10]. FMs have been described in leprosy patients or *M. avium*-infected AIDS (Acquired ImmunoDeficiency Syndrome) patients, and in chronic stages of *M.tb* infection in mice [4],[11],[12]. The foamy aspect of these macrophages is the result of intracellular lipid accumulation within lipid bodies, also called lipid droplets or lipid vacuoles [13],[14],[15]. In an experimental model of leukocyte infection, it was recently suggested that BCG (Bacille Calmette Guerin) infection can induce, in a TLR2-dependent fashion, the rapid formation of lipid bodies carrying out part of the eicosanoïd biosynthesis that usually accompanies the infection, thus pointing to an active role for lipid bodies during the course of infection [16]. However, the mechanisms regulating this lipid accumulation during mycobacterial infection and their significance in the physiopathology of tuberculosis are not understood. Most of the studies on TB granulomas have focused on the contribution of host components, but very little is known about the role played by bacterial constituents in terms of granuloma formation and progression.

The present work was aimed at deciphering the role of FMs in *M.tb* survival within human granulomas. To test our working hypothesis according to which FMs constitute a nutrient-rich reservoir for *M.tb* persistence, we used our in vitro model of human granulomas to analyze the formation of FMs and their role during *M.tb* infection. We showed that only highly virulent mycobacteria (*M.tb*, *M. avium*) and not saprophytic ones (*M. smegmatis*) could induce the formation of FMs in mature granulomas. Moreover, we demonstrated that oxygenated mycolic acids specifically produced by the above pathogenic species were responsible for FMs formation. Once differentiated, FMs were unable to mediate phagocytosis of new bacilli and their microbicidal activity was reduced. *M.tb* was not killed in FMs but instead persisted in a non-replicating state, and over-expressed dormancy genes. Noteworthy, in foamy macrophages, *M.tb*-containing phagosomes were shown to migrate towards lipid bodies which they progressively surrounded and engulfed. As a result, bacteria were freed into lipid bodies, thus favoring the bacilli's access to nutrients. From these data, we propose that FMs could form a secure reservoir for the tubercle bacilli.

## Methods

### Human samples

Human blood samples, purchased from the French National Blood provider of Toulouse, were collected from fully anonymized non-tuberculous control donors, an ethical committee approval was, therefore, not necessary. This study was conducted according to the principles expressed in the Helsinki Declaration, with informed consent obtained from each donor.

We chose to work on lymph node samples rather than lung biopsies, which are usually only paraffin-embedded, because staining for lipids can only be performed on frozen samples. Lymph node biopsies were taken for diagnosis purposes, in ten non-HIV patients. For each biopsy, a fragment was sent to the microbiology laboratory, another was frozen in liquid nitrogen and the main part was fixed in formalin and paraffin-embedded for histological examination. *M.tb* was identified in 9 lymph node biopsies and from the lung aspiration in the last patient. This latter case showed no signs of necrosis, and no FMs were found in the lymph node biopsy. This study complies with the guidelines of the declaration of Helsinki.

### Bacterial strains and culture conditions

Wild-type *M. smegmatis and M. smegmatis/hma* strains were previously described [17], *M. tuberculosis*-GFP were a kind gift from Dr. C. Guilhot (CNRS-IPBS, Toulouse France). Bacilli were grown in Middlebrook 7H9 medium (Difco) supplemented with 10% albumin–dextrose–catalase (Difco). Fluorescent *M. smegmatis* and *M. smegmatis/hma* were obtained by FITC labelling as described in [18].

### Isolation of RNA from intraphagosomal *M.tb*

Six and 12 days post-infection (MOI 10), macrophages and FMs (5×10^6^) were washed twice with PBS, scraped off the cell dishes and recovered by centrifugation. The cell pellets were lysed with lysis buffer (RNEasy mini kit, Quiagen) and transferred to 2 ml Eppendorf-tubes containing a 0.5 ml suspension of 0.1 mm-diameter glass beads (Biospec). Mycobacteria were disrupted using a bead beater (Retsch) followed by a 5 min centrifugation at 14 000g. RNA contained in the supernatant was then column-purified according to the manufacturer's conditions using the RNEasy mini kit (Qiagen) and quantified.

### Quantitative Real-Time RT-PCR

In RNA samples DNA contamination was excluded by DNAse I treatment (Ambion). 1 µg total RNA was reverse-transcribed using random hexamer primers (Ambion) and Superscript III reverse transcriptase (Invitrogen). Real-time PCR was performed on cDNA using the SYBR green essay (Applied Biosystems). Reverse and forward primers used are listed below in Table 1. Fluorescence was measured by ABIPrism 7300 (Applied Biosystems). The calculated threshold cycle (Ct) value for each gene of interest was normalized to the Ct value for 16S and the fold expression was calculated using the formula: fold change = 2−^Δ.^(^ΔCt^) [19]. Real-time PCR conditions include initial activation at 94°C for 5 min, followed by 40 cycles of denaturation at 94°C for 30 sec, annealing and extension at 65°C for 1 min. The gene induction ratios were obtained by comparing gene expression levels in intracellular bacilli with those of log-phase in vitro-grown bacilli. RNAs were isolated from two independent macrophage infections.

**Table 1:** Oligonucleotide primers used in qRT-PCR experiments.

ORF	Sequence *
**Rv2660c**	*GGA ACA GAG GAA CCT TTC GGT G*
	*ACG ATT GAC CTG CGG TTT CA*
**Rv0467**	*CTA CAA CTG CTC GCC ATC GTT C*
	*CAT GGC TGC CAG CTC CTT C*
**Rv3130c**	*ATC CTG ACC AAA CTG CAC CAC*
	*CCC AGC TAG CAG GTG AGT CG*
**Rv3133c**	*GAT GGC AAC GGC ATT GAA CT*
	*ATC AGA CAG CGC AGA TCG G*
**Rv2031c**	*GAA TTC GCG TAC GGT TCC TTC*
	*TGT CGT CCT CGT CAG CAC C*

*: For primers, forward and reverse sequence pairs are listed.

### In vitro human granuloma formation

In vitro granulomas were obtained as previously described [7]. Briefly, 1×10^6^ freshly isolated Peripheral Blood Mononuclear Cells (PBMCs) were incubated with 1×10^4^ viable *M.tb*, or 1×10^3^ viable *M. smegmatis* or *M. smegmatis*/hma. The culture medium was RPMI-1640+Glutamax (Difco), containing 7.5% human AB serum (Sigma-Aldrich).

### Macrophage differentiation

2.5×10^6^ PBMCs prepared in RPMI-1640+Glutamax (Difco) were plated over coverslips in 24-well plates. After 2 h culture at 37°C, cells were washed 3 times with PBS and then refed with RPMI-1640+Glutamax (Difco), containing 7.5% human AB serum. After 6 days of culturing, macrophages were differentiated.

### Respiratory burst assay with Nitroblue tetrazolium (NBT)

Human monocyte derived macrophages were stimulated with *M. smegmatis*/hma for 2 h at 37°C, washed and reincubated in mycobacterium-free medium. After 2 days of differentiation into FMs, macrophages were co-stained with NBT (2 mg/ml Sigma-Aldrich) and Nile red (Sigma-Aldrich) for 30 min at 37°C. Stained cells were fixed and then analysed with an inverted microscope (Nikon TE 300).

### Phagocytosis and Survival test

For phagocytosis assays, differentiated macrophages were incubated with *M. smegmatis*/hma mycolic acids for 2 days and then infected with 1×10^8^ labeled mycobacteria per well for 90 min, washed 3 times with PBS and chased for 3 h in fresh culture medium.

For survival experiments, macrophages were infected with *M. tuberculosis*-GFP (10 bacteria/cell), washed 3 times with PBS (Gibco) and re-incubated in fresh culture medium. At selected time points thereafter (1, 3, 6, 10 and 14 days) cells were labeled with Nile red (Sigma-Aldrich), fixed and observed under a confocal microscope. The amount of mycobacteria per cells was evaluated. (100 cells were analyzed for each time point).

### Lipid body staining and immunostaining

Granuloma cells were collected and plated onto glass coverslips with a cytospin (Thermo Shandon) fixed for 30 min in PBS-PFA 4% and stained with Oil red-O (Sigma-Aldrich) as described [20]. The slides were then counterstained with haematoxylin (Dako Cytomation) and observed under an inverted microscope (Nikon TE 300).

For fluorescence analysis, granuloma cells or macrophages were collected in PBS, lipid bodies were stained with Nile red (Sigma-Aldrich, 0.1 µg/ml, from a stock solution in methanol) for 15 min washed with PBS, fixed for 30 min in PBS-PFA 4%, mounted with the fluorescent mounting medium (Dako Cytomation) and observed under a confocal microscope.

In order to distinguish the lipids contained within lipid bodies, from those of the cell membrane, we used the fluorescent emission spectrum properties of Nile red which depend upon the kind of lipid associated with Nile red, i.e. for triacylglycerol: λmax em = 590 nm, for phospholipids: λmax em = 640 nm (Molecular Probes handbook). On confocal microscopy pictures, the phospholipid background of both macrophages and FM appears in red and the triacylglycerol-rich lipid bodies appear in white. Cells were considered to be positive for Nile red staining when more than 50% of the cell surface was stained (see Figure S2).

### Mycolic acid isolation

Bacterial residues obtained after lipid extraction with organic solvents [17] were saponified with a mixture of 40% KOH aqueous solution and methoxyethanol (1∶7, v/v) at 110°C for 3 hours in a screw-capped tube. After acidification, fatty acids were extracted with diethylether, derivatised into methyl esters with diazomethane and analyzed by analytical thin-layer chromatography on silica Gel 60 (Silica Gel 60 Macherey-Nagel) using either dicholoromethane or petroleum ether/diethylether (9∶1, v/v, five runs). Visualization of lipid spots was performed by spraying the plates with molybdophosphoric acid (10% in ethanol), followed by charring.

### Processing for electron microscopy

Granulomas were fixed for 1 hour at room temperature with 2.5% glutaraldehyde in 0.1 M cacodylate buffer, pH 7.2, containing 0.1 M sucrose, 5 mM CaCl_2_ and 5 mM MgCl_2_. After two successive 15-min washes with the same buffer, the granulomas were postfixed for 1 hour at RT with 1% osmium tetroxide (Electron Microscopy Science) in the same buffer devoid of sucrose. The granulomas were scraped off the culture dishes with a rubber policeman and concentrated in 1% agarose in the same buffer. After a one hour treatment at room temperature with 1% uranyl acetate in Veronal buffer, the samples were dehydrated in a graded series of ethanol and embedded in Spurr resin. Thin sections were stained with uranyl acetate and lead citrate.

### Image acquisition in confocal microscopy

The images were obtained using a Leica confocal fluorescence microscope (SP2) equipped with a Plan Apo 40×1.4 Ph 6 objective (Olympus Optical) and CoolSNAP-Pro CF digital camera in conjunction with Image-Pro Plus version 4.5.1.3 software (Media Cybernetics). The images were edited using Adobe Photoshop CS2 9 software (Adobe Systems).

## Results

### Foamy macrophages are strongly associated with necrotic lesions and often contain *M. tuberculosis*

**Figure 1:**
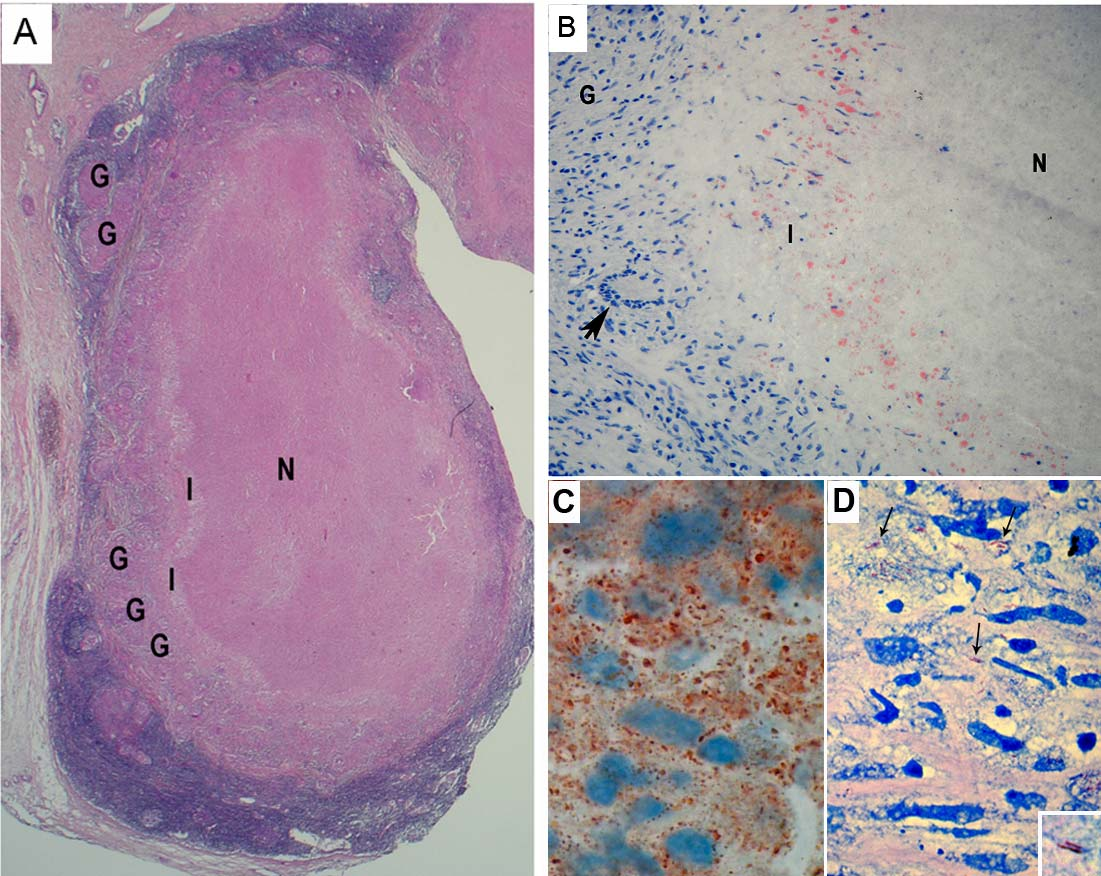
Granulomas from TB patients display many *M.tb*-containing FMs surrounding the necrotic center. Thin sections of lymph node biopsy samples from 10 tuberculous patients were stained and analyzed. A) Haematoxylin and Eosin staining, original magnification ×12. B, C) Oil red-O staining, original magnification ×200 (B) and ×1000 (C). D) Ziehl-Nielsen staining (×1000). G: granuloma, N: necrosis, I: interface. Arrow: Giant Langhans cell (B), *M.tb* (D).

We analyzed lymph node biopsies from 10 tuberculous patients as a first step for evaluating the role of FMs within tuberculous granulomas. A section through a representative biopsy is shown in Figure 1. Well-circumscribed and -differentiated granulomatous structures were observed in all the samples (Figure 1A). Classically, lesions display a necrotic center (N), an interface area between the necrotic center and the histiocytes (I), and some peripheral granulomas (G). Only seven out of ten patients presented lesions displaying central necrosis (Table 2). Staining of the histology samples with Oil red-O, a classic lipid stain, confirmed the presence of FMs within the granulomatous structures in six out of seven samples presenting necrosis (Figure 1B), whereas no FMs were found in the three non-necrotic lesions (Table 2). Noteworthy, in samples with necrotic areas, FMs were always found in the interface region flanking the central necrosis (Figure 1B). These observations firstly confirmed the presence of FMs in most TB patients' lesions thereby suggesting that these cells play an important role in the formation/maintenance of such lesions. Second, FMs seem to be associated with necrosis, which is a hallmark of TB lesions, since they were observed only in lesions with a necrotic center and preferentially located around the necrotic area.

**Table 2:** Lymph node biopsies from 10 tuberculous patients were analysed for the presence of necrotic lesions and stained with Oil red-O to assess the presence of FMs.

Age	sex	Necrosis	FM
20,3	M	+	+
50,5	M	−	−
50,4	F	+	+
27,2	F	+	−
12,9	F	+	+
77,1	F	−	−
82,7	M	−	−
79,0	F	+	+
27,8	F	+	+
88,4	F	+	+

Interestingly, staining, in parallel, of serial thin sections from a patient's lesion biopsy with Oil red-O (Figure 1C) and Ziehl Nielsen (Figure 1D), showed that most of the bacilli (arrow) were located in the same area as FMs, thus suggesting a strong association between the persisting tubercle bacilli and FMs within granulomas.

### *M. tuberculosis* induces the formation of FMs within in vitro human tuberculous granulomas

**Figure 2:**
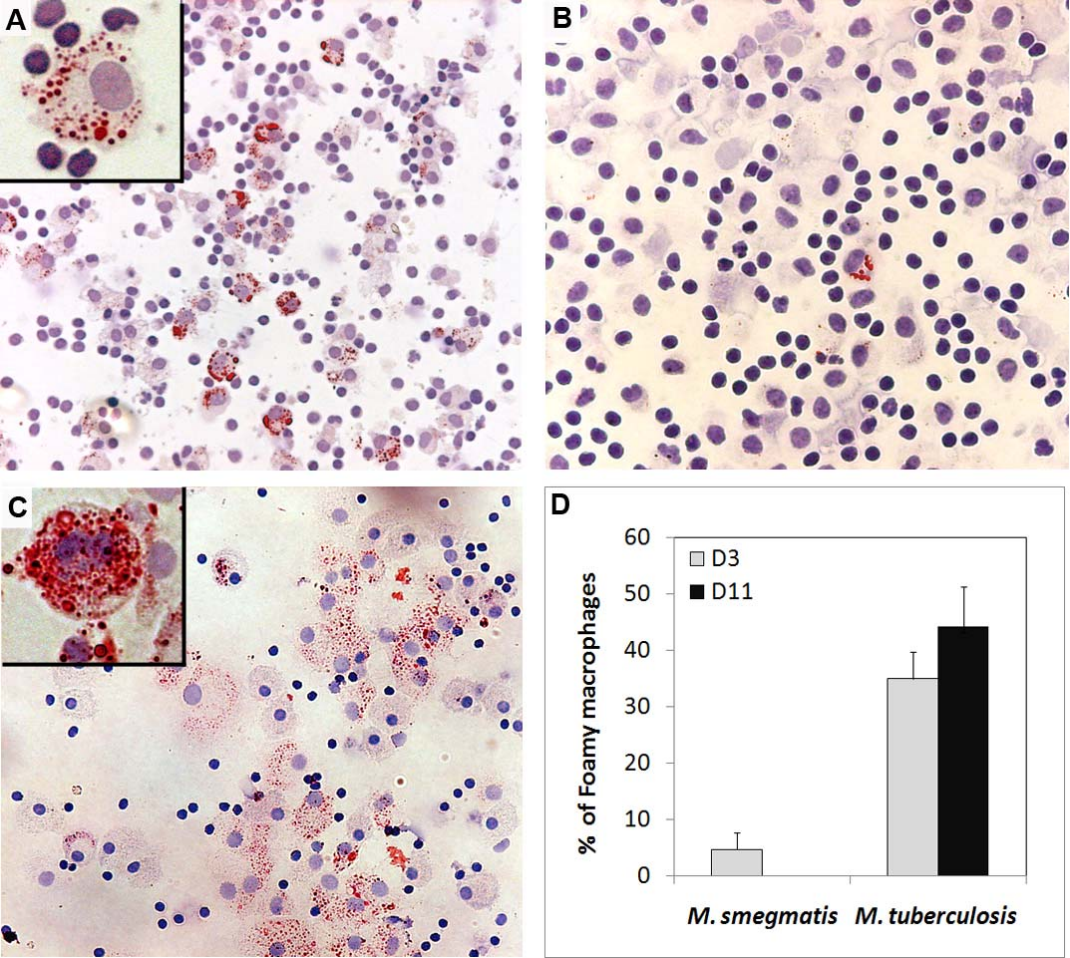
*M.tb* but not *M. smegmatis* induces FM formation within in vitro granulomas. PBMCs from a healthy donor were infected with *M.tb or M. smegmatis*. Granuloma cells were collected 3 (A, B) and 11 (C) days later and stained with Oil red-O. The percentage of FMs within the whole macrophage population is indicated in panel D. The original magnification for the general views is ×100 (A, B), and ×200 (C), ×1000 for the enlarged views. These pictures are representative of 5 independent experiments with 5 unrelated controls.

To further characterize the role of FMs in the granulomatous response, we assessed whether FM formation in granulomatous structures was triggered only by pathogenic mycobacterial species (*M.tb*), or by low virulent ones (*M. smegmatis*) as well. PBMCs from non-tuberculous control individuals were infected with *M.tb* or *M. smegmatis*, following the procedure previously described for the induction of granulomatous structures ([7],[9] and Figure S1). Granuloma cells collected at days 3 and 11 were stained with Oil red-O to visualize the lipid droplets within FMs under the light microscope (Figure 2). At day 3, several *M.tb*-induced granuloma cells already showed lipid bodies (Figure 2A). In contrast, the cells collected from *M. smegmatis*-induced granulomas were seldom (5%) positively stained (Figure 2B). Interestingly, *M. avium* induced FM formation in a similar way to *M.tb* (not shown). By day 11, the amount of positively stained cells had increased in *M.tb*-induced granulomas, but not in *M. smegmatis*-induced ones. In addition, the number of lipid bodies per cell increased dramatically with time, as depited in the enlarged views (Figures 2A, C). The quantitative evaluation of the percentage of FMs within granulomas induced by both strains confirmed the differences observed under the light microscope, and showed a seven-fold difference (44% vs 6% respectively) between *M.tb* and *M. smegmatis* in terms of their ability to induce FM formation (Figure 2D). Our results therefore show that virulent species such as *M.tb* and *M. avium*, contrary to poorly or avirulent ones such as *M. smegmatis*, are able to induce the formation of FMs within our experimental model.

### Oxygenated mycolic acids induce the maturation of macrophages into FMs

**Figure 3:**
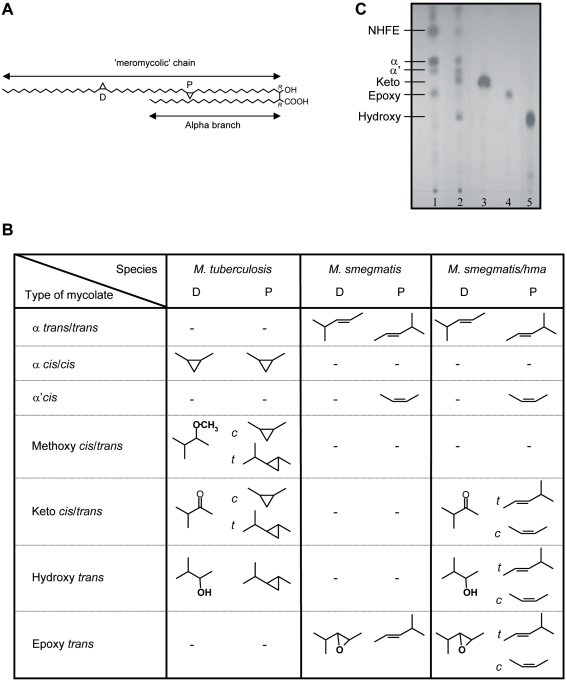
Structure of the mycolic acids found in *M.tb*, *M. smegmatis and M. smegmatis/hma* respectively. A) Dicyclopropanated mycolic acid (α-mycolate) from *M. tuberculosis*. The chemical functions introduced by methyltransferases occur at the proximal (P) and distal (D) positions. B) TLC profile of the mycolic acid methyl esters from: lane1, *M. smegmatis*; lane 2, *M. smegmatis/hma*; lane 3, ketomycolate; lane 4, epoxymycolate; lane 5, hydromycolate. NHFE, non-hydroxylated fatty esters. C) Types of functional groups present at both the proximal and distal positions of the different species of mycolates found in *M. tuberculosis*, *M. smegmatis* and *M. smegmatis/hma*.

Mycolic acids from *M.tb* incorporated into liposomes were recently shown to trigger the differentiation of mice peritoneal macrophages into foamy-like cells [21]. Interestingly, both *M.tb* and *M. avium*, which induce FM formation, express a family of oxygenated mycolic acids, especially ketomycolic acids, which are not produced by *M. smegmatis* (Figure 3A). In this context, inactivation of the *M.tb hma* gene (*mmaA4*-Rv0642c) was shown to abolish the synthesis of oxygenated keto- and hydroxyl-mycolic acid in the mutant strain [17]. Conversely, transforming *M. smegmatis* with the *hma* gene induced the production of both keto- and hydroxyl-mycolic acids [22], (Figures 3B, C).

**Figure 4:**
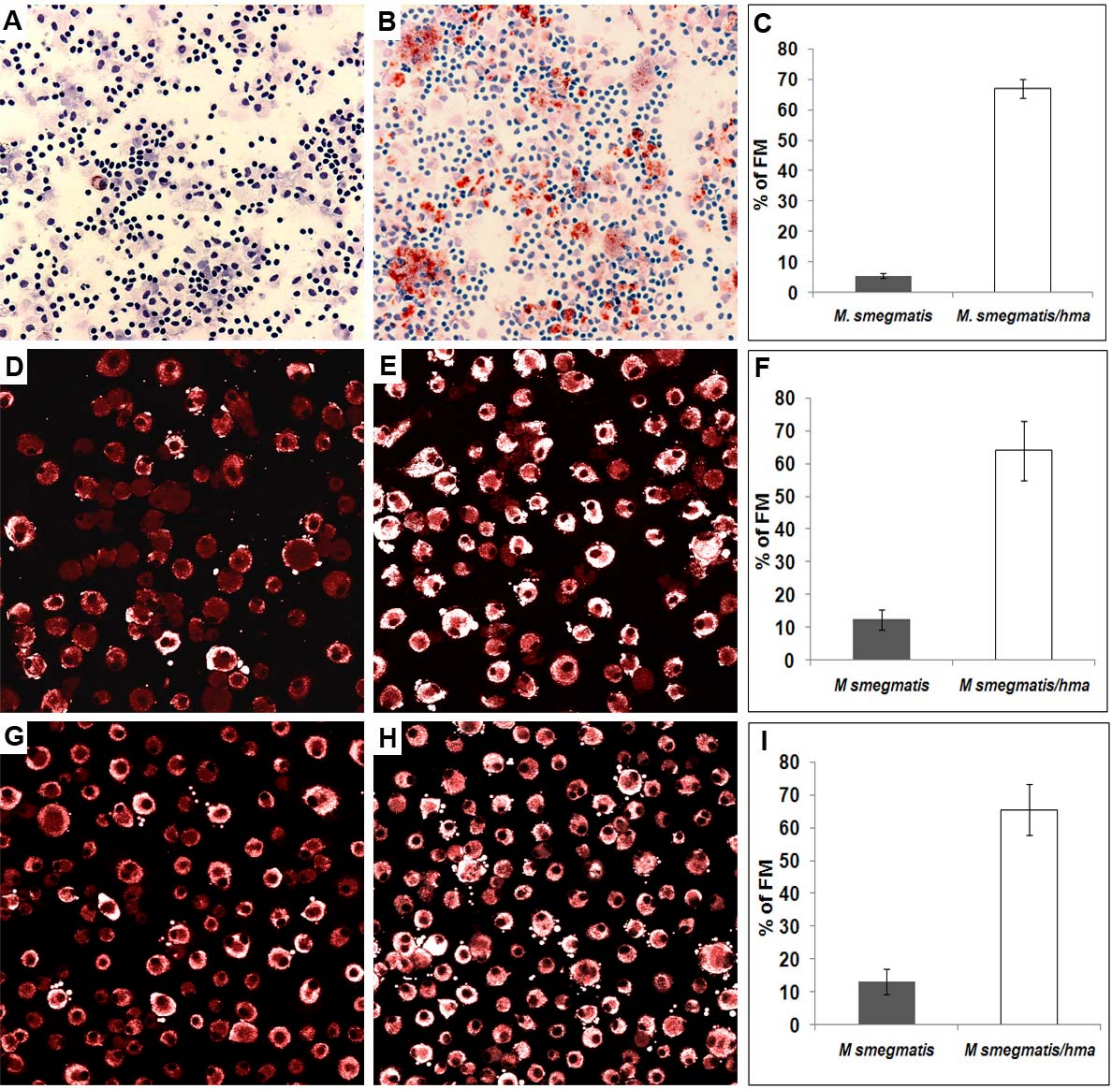
Role of *hma*-controlled mycolic acids in FM formation. PBMCs from a healthy donor were infected with either *M. smegmatis* or *M. smegmatis/hma* for 3 days. The granuloma cells were then collected and stained with Oil red-O and May-Grünwald Giemsa (A, B). In parallel experiments, isolated macrophages were infected with either *M. smegmatis* (D) or *M. smegmatis*-hma (E), or incubated with mycolic acids extracted from both species (*M. smegmatis* G, *M. smegmatis*-hma H). The cells were then stained with Nile red. The pictures are representative of 3 independent experiments with 3 unrelated controls. Original magnification: A, B: ×100; D, E, G, H: ×400. The percentage of FMs in the different macrophage populations are indicated in panels C, F, I, respectively.

In the light of both data, we anticipated that oxygenated mycolic acids specifically produced by *M.tb* and *M. avium*, under the control of *hma*, are responsible for FM formation within human granulomas. To test this hypothesis, we compared FM formation after infection of PBMCs with either the wild-type, or the hma-expressing *M. smegmatis* strain (*M. smegmatis*/hma). Granulomas cells were collected 3 days later and stained with Oil red-O to visualize lipid bodies. Wild-type *M. smegmatis*-induced granulomas displayed only 5.5% of FM, whereas the *hma* gene-expressing strain induced granulomas bearing a majority (67%) of brightly stained Oil red-O positive cells (Figures 4A, B, C). Induction of FM formation was even greater if isolated macrophages were directly infected with either strain. After only 4 hours of infection, *M. smegmatis*/hma had already transformed 64% of the infected macrophages into lipid body-positive cells (see Figure S2 for Nile red positive cells), whereas only 9% of the cells contained lipid bodies after infection with the wild-type strain (Figures 4D, E, F). To confirm the specific role of *hma*-dependent oxygenated mycolic acids in FM formation, and to rule out a possible combined effect of oxygenated mycolic-acids with other mycobacterial components, mycolic acids isolated from wild-type *M. smegmatis* or *M. smegmatis*/hma were incubated with isolated macrophages. With mycolic acids isolated from the wild-type strain, only 13% of the macrophages were transformed into FM whereas 66% of the macrophages incubated with mycolic acids isolated from *M. smegmatis*/hma were strongly stained for lipid bodies (Figures 4G, H, I).

These results therefore indicate that oxygenated mycolic acids play a leading role in *M.tb*-induced FM formation.

### The phagocytic and bactericidal activities are arrested in FMs

**Figure 5:**
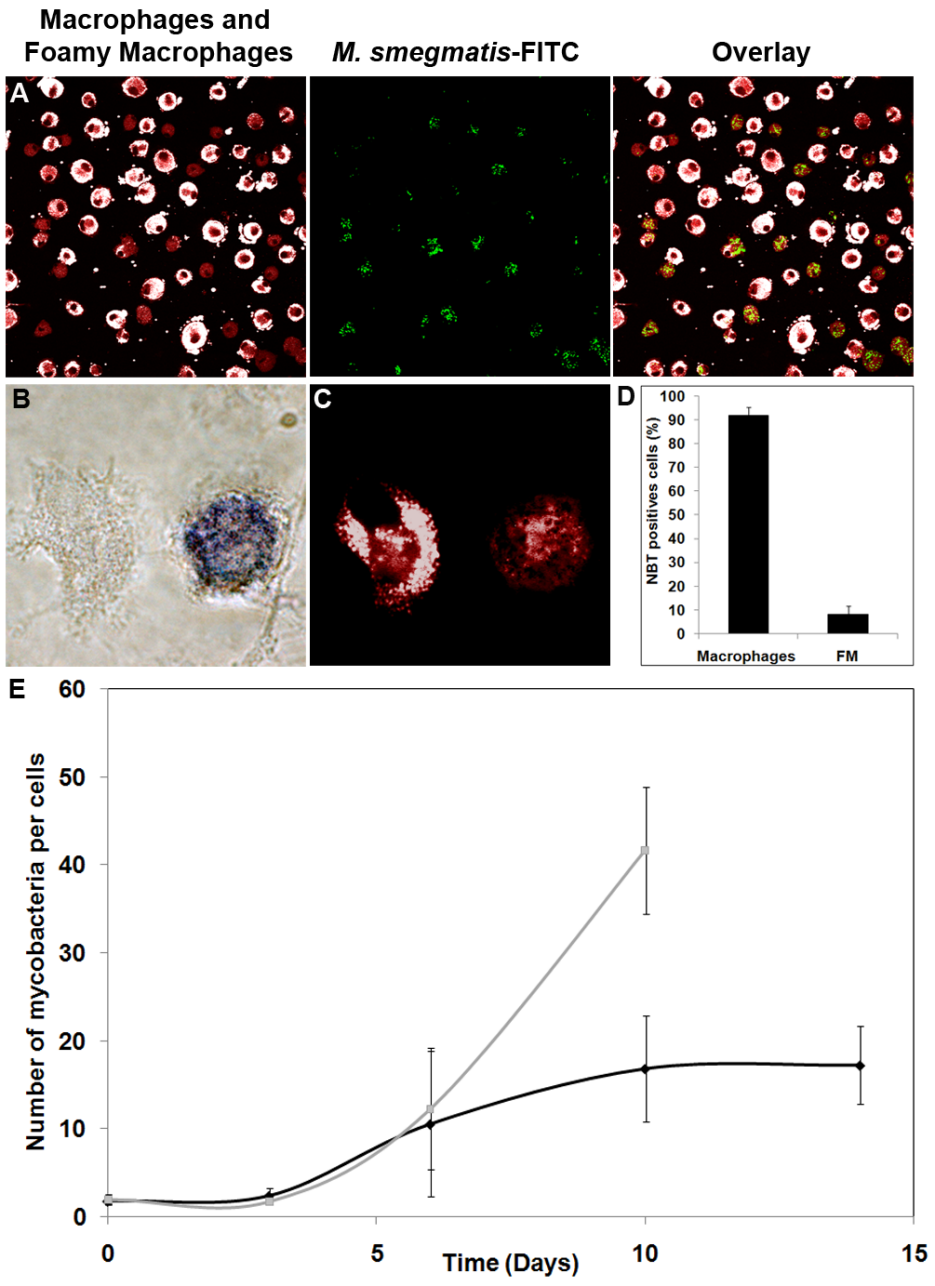
Phagocytic uptake and survival of mycobacteria within FMs. A) Isolated macrophages from a control donor were incubated for 2 days with *M. smegmatis/hma*-isolated mycolic acids, and then infected with FITC-labelled *M. smegmatis* for 1 hour. The cells were then stained with Nile red. Original magnification ×400. Isolated macrophages from a control donor were infected with *M.tb* for 2 days, and then stained with Nile red (B, ×1000) and subsequently analyzed for NBT reduction ability (C, ×1000). The proportion of FMs (Nile red positive cells) able to reduce NBT was then evaluated (D). E) Macrophages from a healthy donor were infected with GFP-expressing *M.tb* for 1 hour, washed and re-incubated in fresh medium. At selected times after infection, the number of bacteria per cell was determined under a confocal microscope for both macrophages (Nile red-negative cells) and FMs (Nile red-positive cells). The data are representative of 3 independent experiments. In order to distinguish the lipids contained within lipid bodies, from those of the cell membrane, we made use of the fluorescent emission spectrum properties of Nile red which depend upon the lipid which is associated with Nile red, *i.e.* for triacylglycerol (λmax em = 590 nm), for phospholipids (λmax em = 640 nm) (Molecular Probes handbook). On confocal microscopy pictures, the phospholipids background of both macrophages and FMs appears in red, and the triacylglycerol-rich lipid bodies in white. Cells were regarded as positively stained by Nile red when more than 50% of the cell surface was stained (see Figure S2).

To assess the function of granuloma FM, we first evaluated the ability of such cells to mediate phagocytosis. For this purpose, macrophages isolated from PBMCs were exposed to *M. smegmatis*/hma-derived mycolic acids to induce FM formation. Two days later, the cell population contained a mixture of FM (50–70%) and macrophages (30–50%), as assessed by Nile red staining (not shown). The mixed cell population was infected with FITC-labelled *M. smegmatis*. Intracellular bacilli were found only within Nile red-negative macrophages thereby indicating that FMs are unable to ingest bacteria (Figure 5A). This result was reproduced using other mycobacterial strains, such as *M.tb* and *M. bovis* BCG, for infection (not shown). These results further suggest that the bacilli found in granuloma FMs were internalized by macrophages prior to their transformation into FMs.

To assess whether FMs are able to develop a respiratory burst, which is a major intracellular bactericidal activity, the ability of Nile red positive cells (*i.e.* FMs) to mediate NBT reduction was determined. As shown in Figure 5D, only 8% of the NBT-positive cells (Figure 5B) were Nile red positive FMs (Figure 5C). This strongly suggests that once macrophages have differentiated into FMs, they lose the ability to mediate intracellular bactericidal activity. We postulate that FMs could, therefore, form a secure reservoir for the tubercle bacilli.

### *M. tuberculosis* persists in a dormant non-replicative state in FMs

To evaluate the validity of the above hypothesis, we analyzed the ability of *M.tb* to replicate within FMs. For this purpose, isolated macrophages were infected with *M.tb*. At selected intervals post-infection, the amount of bacilli per cell was compared in both non-differentiated macrophages and FMs (Figure 5E). Until day 6, *M.tb* replicated in a similar fashion in both cell types. In contrast, after day 6 post-infection, the amount of bacilli remained stationary in FMs, whereas it continued to increase in macrophages. It is interesting to note that arrest of bacterial replication coincided with completion of macrophage differentiation into FM, *i.e.* starting from day 3 post-infection. Our data suggest, that the bacilli found in granuloma FMs were internalized by macrophages prior to their differentiation into FMs and also that bacilli can terminate their replication cycle while macrophages are being transformed into FM, but that replication comes to a halt as soon as the maturation process is complete.

We next determined whether the non-replicative bacilli observed in FMs were still alive. For this purpose, we analyzed the expression of a series of genes known to be up-regulated when bacilli are in a persistent non-replicating state [23],[24]. RNA was, therefore, prepared from both in vitro-grown *M.tb* and intracellular bacilli at day 6 and 12 post-infection. The respective amounts of RNA corresponding to isocitrate lyase, α-cristallin, a very hypothetical 7.6 kDa protein, CHP and DosR proteins were then quantified by RT-PCR. As shown in Table 3, the dormancy genes were all strongly up-regulated in intracellular bacilli at day 12 post-infection. These results further demonstrate that the bacilli are not killed in FMs, but rather persist in a dormant, and therefore non-replicative stage [25]. Interestingly, the dormancy genes were not as strongly expressed at day 6, time at which bacteria were still able to replicate in macrophages undergoing differentiation into FMs.

**Table 3:** RT-PCR analysis of dormancy gene expression from intracellular *M. tuberculosis* at 6 and 12 days post-infection.

ORF	Gene name	Protein function	Fold induction	
			Day 6	Day 12
**Rv0467**	*AceA*	isocitrate lyase	158.99	261.17
**Rv2031c**	*hspX/acr*	α-crystallin	ND	720.26
**Rv2660c**	-	very hypothetical 7,6 kDa protein	0.13	6.96
**Rv3130c**	-	CHP	0.11	116.54
**Rv3133c**	*dosR*	2-comp. Response reg.	4.4	16.67

ND: Not Detected.

RNA was extracted from macrophages infected with *M. tuberculosis*. Each point represents *M. tuberculosis* dormancy gene expression ratios in macrophages 6 and 12 days after infection as compared with that of log-phase in vitro-grown *M. tuberculosis*.

### Characterization of *M.tb* survival within FMs and interaction with lipid bodies

To gain further insight into the morphological appearance of *M.tb* within FM and into the interactions between FM lipid bodies (LB) and *M.tb*-containing phagosomes, granulomas were fixed and processed for conventional electron microscopy at days 3 and 11 post-infection.

**Figure 6:**
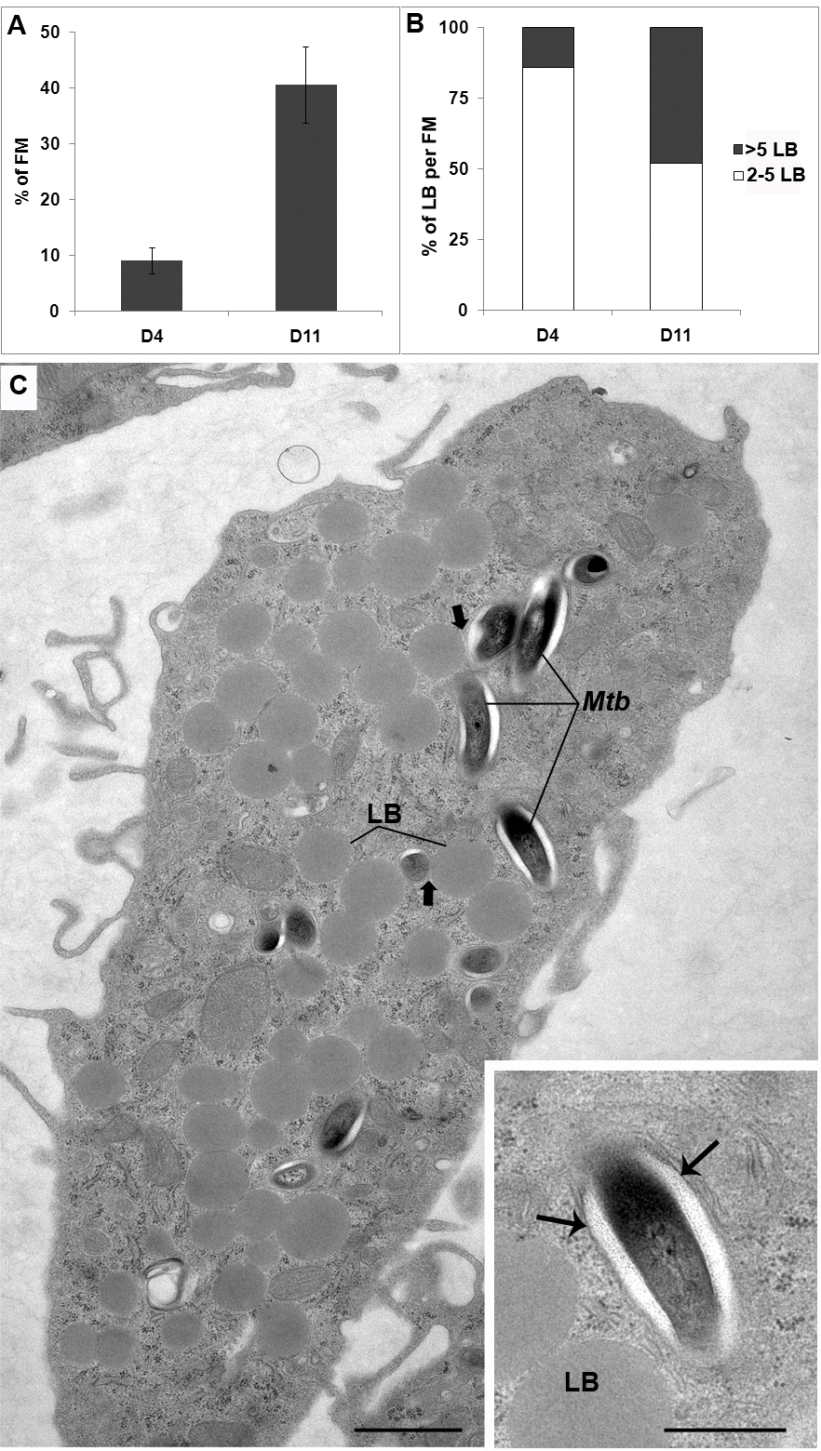
Morphological appearance of foamy macrophages and bacteria within granulomas. PBMCs were infected with *M.tb* H37Rv, fixed and processed for electron microscopy at days 3 and 11 post-infection. A) The percentage of FM, with respect to the total amount of macrophages encountered on thin sections of granulomas, was determined. Care was taken to avoid serial sections. B) The number of LBs encountered in the above FMs was next determined. Results are expressed as the percentage of FMs, with respect to the total number of FMs, displaying either 2 to 5 LBs (□) or more than 5 LBs (▪). C) General view of a FM containing a large number of lipid bodies (LB) at day 11 post-infection. In this cell, some of the *M.tb*-containing phagosomes are in the close vicinity of a LB (large arrow). Enlarged view of one bacterium within a membrane-bound phagosome (small arrows) showing the ultrastructural integrity of bacteria within foamy macrophages and the lack of bacterial intracytoplasmic lipid bodies (ILI). Bar: C) 1 µm; D) 0.5 µm.

Whatever the time point at which granuloma cells were observed, bacteria were all enclosed in phagosomes, most of which contained a single bacterium. None of them (over a thousand which were examined under the electron microscope) were free in the cytoplasm and only one was enclosed in a classical autophagic vacuole. At day 3 post-infection, FMs profiles (thin sections) were scarce, representing at most 9% of the total population of macrophage profiles observed under the electron microscope (Figure 6A). In addition, 86% of the FM profiles displayed at most 5 small LBs (Figure 6B). At this stage of granuloma formation, bacteria were infrequent in FM, but were found in other types of macrophages. One of these displayed large numbers of vacuoles containing flocculent material and often one or two LBs. Over 95% of the bacteria located in the different granuloma macrophages were morphologically intact, and therefore alive [26]. Intact bacteria present no breaks in the cell wall or cytoplasmic membrane and their cytoplasm has preserved its ultrastructural organization and electron opacity. Furthermore, they display no electron translucent intracytoplasmic lipid inclusions (ILI). Bacteria were also observed in between cells, probably as a result of cell lysis within granulomas. These bacteria were also morphologically intact and devoid of ILI (not shown).

At day 11 post-infection, *M.tb*-containing macrophages displaying large numbers of vacuoles with flocculent material were less frequently observed. Interestingly, the amount of such vacuoles had strongly decreased in most of these cells while the number of LBs had increased. The percentage of FM had increased to reach 41% of the total population of macrophage profiles within the granulomas (Figure 6A). From these observations, it is tempting to assume that the highly vesiculated macrophages give rise to FMs. Within FMs, the size and amount of LBs had also increased with time since 48% of the FM profiles now displayed more than 5 LB per FM thin section (Figure 6B), randomly distributed within the cells (Figure 6C). About 30% of the FM profiles displayed between 1 and 20 bacteria, which were morphologically intact and enclosed within phagosomes (Figure 6C, enlarged view).

**Figure 7:**
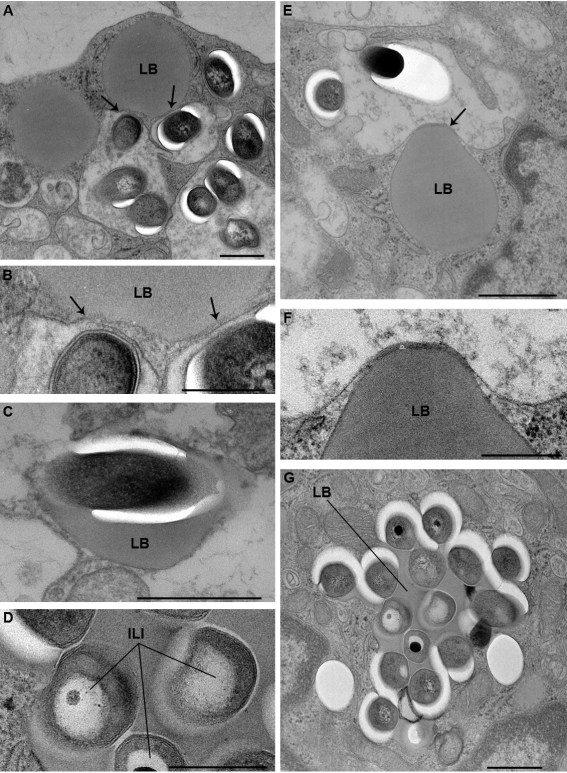
Interaction between cellular lipid bodies (LB) of foamy macrophages and *M.tb*-containing phagosomes. PBMCs were infected with *M.tb* H37Rv, fixed and processed for electron microscopy at day 11 post-infection. A) shows 3 *M.tb*-containing phagosomes which are closely apposed to a LB (arrows); B) enlarged view of part of the phagosomes in A, showing the tight contact between the phagosome membrane and the LB (arrows); C) *M.tb* in a LB. This bacterium displays no intracytoplasmic lipid bodies (ILI); D) enlarged view of *M.tb* within the LB depicted in G. These bacteria contain large ILI. E) shows a *M.tb*-containing phagosome, tightly apposed to a LB, and which is starting to engulf this LB; F) Enlarged view of part of E showing the tight apposition of the phagosome membrane to the LB; G) LB containing several *M.tb*. Some of the bacteria now display ILIs (see D for detailed view of these bacteria). Bar: A, C, E, G) 0.5 µm; B, D, F) 0.25 µm.

The interaction between these bacteria and the cellular LBs was next examined at day 11. Sixty percent of the bacilli were scattered throughout the FMs and displayed no obvious signs of interaction with the cellular LBs. A small fraction of the bacteria (21%), however, were observed in the close vicinity of cellular LBs. The membrane of the phagosomes in which they were enclosed clearly interacted with cellular LBs (Figures 6C, 7A, 7B, arrows) and became tightly apposed to an increasingly larger surface area of the LB. As a result, the phagosomes started to surround LBs in a zippering fashion (Figures 7E, F). Ultimately, bacilli (19%) were translocated to cellular LBs (Figure 7C). From these observations, it is tempting to assume that *M.tb*-containing phagosomes engulf cellular LBs rather than fusing with them. This process, which is reminiscent of autophagy, resulted in the transfer of free bacteria into the lumen of cellular LBs (Figure 7C), some of which displayed up to 21 bacteria (Figure 7G). Interestingly, only altered *M.tb* found within FM lipid bodies exhibited electron translucent ILIs (Figures 7D, G), thereby suggesting that they are able to accumulate host cell lipids. In previous work, the term altered bacteria had been used to define live bacteria that had acquired ILIs [26]. Since the presence of ILIs within the cytoplasm of *M.tb* is typical for non-replicating bacteria in a state of dormancy [27], this further confirms that these bacteria are dormant.

## Discussion

Studies carried out several decades ago suggested that postprimary tuberculosis starts as a lipid pneumonia [28],[29]. Indeed, following a first inflammatory process leading to exudates of mononuclear cells within alveolar spaces, early pathologists observed an accumulation of lipid droplets in alveolar macrophages of TB patients. Tubercle bacilli were shown to reside in these lipid-rich macrophages which were named foamy macrophages (FMs) [30]. Recently, histo-pathological analysis of biopsies from patients with untreated tuberculosis confirmed the century-old histological descriptions of postprimary tuberculosis [10]. In this study, Hunter *et al* showed that postprimary tuberculosis begins as a lipid pneumonia with the accumulation of large amounts of lipid-rich FMs, accompanied by bronchial obstruction. It was also shown that in alveolar foamy macrophages, the bacilli were mainly found within lipid droplets. All these observations underline the important, yet often neglected, role of lipid accumulation, and more precisely FM formation, at the infectious site in the physiopathology of TB.

In murine experimental models, FMs accumulate within the outermost layer of granulomatous structures occupying the alveolar spaces during the chronic phase of infection. This strongly suggests that FMs could be involved in lesion cleaning via phagocytic uptake of cellular debris generated by the local inflammatory response. Once filled with debris, FMs would leave the parenchyma through the alveolar spaces up to the superior bronchial tree, to be finally swallowed and digested in the stomach [31]. In fact, this process is very well known, and is a crucial factor for TB diagnosis in children. As infants do not usually generate cavitary lesions, and because it is difficult to detect bacilli in the sputum, the diagnosis is linked to the detection of bacilli in the gastrointestinal lavage [32].

We show that *M.tb*-induced the transformation of in vitro-grown human granuloma macrophages into FMs within 6 days, and even more rapidly (3–4 hours) in cultured macrophages. Although this event occurs more quickly than in vivo, or in animal models, our data are consistent with the above in vivo observations. Within FMs, bacilli and LBs were often tightly linked, to the point that a non-negligible amount of bacteria were ultimately observed within LBs. Interestingly, some of the bacilli transferred into lipid bodies displayed their own intracytoplasmic lipid inclusions, which are considered to be one of the hallmarks of non-replicating (dormant) *M.tb* [27]. The recent observation of persistent ILI-containing tubercle bacilli within adipocyte LBs [33] is in good agreement with our observations. Since bacilli residing in phagosomes that do not interact with cellular lipid bodies do not display ILIs, it is tempting to assume that lipids within ILIs are of cellular origin. The accumulation of lipids within bacilli [47], via interaction with FM lipid bodies could, therefore, be crucial to *M.tb* persistence. It is indeed known that *M.tb* accumulates lipids, and more precisely triacylglycerols, during dormancy [34],[35] from which it derives both carbon and energy for its own metabolism. Intracellular persistence of *M.tb* is also critically linked to the acquisition of host cholesterol through the Mce4 transporter system [36].

The question that arises is how do bacteria gain access to lipids from LB? Direct fusion of phagosomes with FM lipid bodies seems unlikely as the membranes of both structures are quite different from one another. Our observations suggest instead that once *M.tb*-containing phagosomes have established close contact with a lipid body, they surround and engulf the latter by a process that remains to be deciphered. This phenomenon is somewhat reminiscent of autophagy, as observed under conditions of cholesterol depletion in macrophages infected with *M. avium* [37]. After degradation of the resulting inner membrane, bacteria would be freed within the lipids of the engulfed LB, and therefore be in direct contact with cellular lipids. How bacilli translocate the cellular lipids to their own cytoplasm remains to be established.

Another important phenomenon underlined by our study is the strong correlation between the presence of FMs in the granulomatous structures and the development of necrosis within the lesion, as suggested by Pagel over 80 years ago [29]. Interestingly, FMs were systematically located at the interface region between the histiocytes and the central necrosis area of the biopsied lesions. Although necrosis formation could depend on an indirect effect of the global immune response, our data indicate that the formation of FMs is an important factor favoring the appearance of necrosis. Analysis of larger series of biopsy samples are, however, needed to definitely demonstrate our actual hypothesis according to which FMs play a direct and unique role in necrosis formation. Consistent with this hypothesis, we observed that FMs induced from *M.tb*-infected macrophages displayed permanent TNF-α secretion, a potent pro-necrotic factor, whereas *M. smegmatis*-infected macrophages were poor producers of TNF-α. At day 4 post-infection, TNF-α secretion was indeed twofold higher in macrophages (of which 70% had differentiated into FMs) infected with *M.tb* than in those infected with *M. smegmatis*, as measured both by ELISA and RNA quantification (Peyron, unpublished observations). However, one must keep in mind that the association of FMs and necrosis may be the consequence of the FM cleaning process of lipoproteins released into the necrotic tissue, as observed in atherosclerosis lesions [38]. It is thus tempting to propose that *M.tb* mycolic acids may be responsible for the development of necrotic lesions, due to their ability to induce TNF-α production by FMs. Whether mycolic acids are directly involved in TNF-α production, or only indirectly by inducing FM formation, remains a matter of debate currently under study.

Our observations are strikingly similar to the phenomena described for postprimary tuberculosis, that seems to begin as localized foci of pneumonia followed by massive necrosis leading to the formation of pulmonary cavities [10]. If this proves to be the case, then the traditionally admitted phenomenon of cavitation arising from the erosion of caseating granulomatous structures from bronchi can be ruled out [39]. In our study, we successfully induced FM formation from isolated macrophages infected with *M.tb*, *i.e.* outside a granulomatous structure, which is consistent with Hunter's recent proposal.

Until now, mycolic acids have been considered to be indirectly involved in virulence mechanisms as being part of complex molecules of the mycobacterial envelope. The most widely studied mycolic acid-containing mycobacterial compound trehalose 6,6′ dimycolate (TDM), has been extensively analyzed for its role in virulence since the mid-fifties [40]. Recently, it was shown to interfere with the host granulomatous response [41]. Overall, TDM was mainly shown to mediate macrophage activation and a Th1-type response to *M.tb* infection (for review, see [25]).

Our results demonstrate a direct role of oxygenated mycolic acids for FM formation, independently from the appearance and stage of the disease. *M.tb*-specific mycolic acids indeed trigger the transformation of both isolated and granuloma macrophages, into FM. Given the absence of FM formation in *M. smegmatis*-induced granulomas, despite the induction of a comparative inflammatory response, ascertained by the similar induction of granulomas, this phenomenon clearly depends upon a direct contact with the bacilli, and not to the inflammatory response. Mycolic acids are major and hallmark components of the mycobacterial cell wall. They constitute 40–60% of dry weight of the envelope [42]. All members of the complex (*e.g. M.tb*, *Mycobacterium africanum*, *Mycobacterium bovis and Mycobacterium microti*) are able to synthesize the same combination of mycolic acids, i.e. cyclopropanated α-mycolic acids, ketomycolic and methoxymycolic acids [17], which are not synthesized by non-pathogenic mycobacterial species [43]. These structural specificities probably account for part of the pathogenicity of these species, as shown by the impaired virulence of mutant strains deprived of keto and methoxyl groups in experimental infections [17],[44],[45].

Our study, therefore, gives the first proof of a direct role of isolated mycolic acids in the interplay between *M.tb* and host cells. Interestingly, this effect is expressed both by whole bacilli and isolated lipids, suggesting that oxygenated mycolic acids are either secreted by the bacilli, or exposed at the cell wall surface in a manner enabling their bioactivity. According to our results, mycolic acids trigger the formation, within granulomas, of FMs in which bacilli can hide and survive. Oxygenated mycolic acids, either free, as constituents of TDM [44], or linked to the cell wall arabinogalactan [46],[47], should, therefore, be considered as major virulence factors enabling *M.tb* survival for long periods of time in a persistent state. Being an inducer of host lipid accumulation, and FM formation at the site of infection, these oxygenated mycolic acids could, therefore, also be responsible for the induction of necrosis within lesions, thus favoring *M.tb* dissemination.

Interestingly, deletion of the *mmaA4* (Rv0642c) gene also drastically decreased the ability of *M.tb* to induce the differentiation of macrophages into FMs (data not shown). However, the residual ability of this mutant to induce FMs suggests that other mycobacterial factors might partially trigger the formation of FM. With regard to the mycolic acid methyltransferases, given that (i) *mmaA2* (Rv0644c) and *mmaA3* (Rv0643c) are pseudogenes in *M. leprae* and (ii) *mmaA4* KO present no trans cyclopropanation [45], thus excluding the involvement of the *cmaA2* (Rv0503c) gene, we expect that at least *pcaA* (Rv0470c), which introduces cis-cyclopropane, may play the same role. Consistent with this hypothesis, a *pcaA* null mutant is unable to persist within infected mice [46], thus demonstrating the role of a mycolic acid methyltransferase in the chronic stage of infection.

Overall, our study has shed light on a previously uncharacterized cell population participating in human tuberculous granulomas, namely foamy macrophages. We propose that the specific induction of FM by *M.tb* would create a favourable environment for persistent bacteria. In our opinion, FMs could be a safe shelter because they preserve bacilli from a direct contact with granuloma lymphocytes and histiocytes, they lose one of the major macrophage bactericidal activities and they constitute an important source of nutrients for the bacilli thanks to the fatty acids accumulated in their lipid granules.

## Supporting Information

Figure S1 In vitro-induced *M.tb* granulomas strongly mimic in vivo granulomas. A. Scanning electron microscope observation of a *M.tb* granuloma 9 days post-infection. The structure of the granuloma is well-defined and -circumscribed, and both macrophages and lymphocytes can be observed around the structure, as is typical of a human granuloma. Bar: 50 µM. B. Peripheral lymphocytes were removed from day 11 granulomas by smooth pipeting, and the remaining structure was stained with Oil red-O and haematoxylin. As in the case of lesion biopsies, a large number of FMs (red staining) can be observed around the central area of in vitro granulomas. Original magnification ×100. C. Confocal microscopy analysis of granulomas induced by a GFP-expressing *M.tb* strain. To outline the cell contour, the cells were permeabilized and labelled with β-Phaloïdin that stains cortical actin filaments. Original magnification ×400.
(2.93 MB TIF)

Figure S2 Nile red staining. In order to distinguish the lipids contained within lipid bodies from those of the cell membranes, we took advantage of the fluorescent emission spectrum properties of Nile red which depend upon the lipid Nile red is associated with, i.e. for triacylglycerol: λmax em = 590 nm, for phospholipids: λmax em = 640 nm (Molecular Probes handbook). On confocal microscopy pictures, the phospholipid background of both macrophages and FMs appears in red and the triacylglycerol-rich lipid bodies of FMs appear in white. Cells were considered to be Nile red-positive when more than 50% of the cell surface was stained. Using this criterion, the bottom right cell is a FM and the cell in the upper left corner a macrophage.
(1.06 MB TIF)
