# Supplementary figures and images for: Foamy Macrophages from Tuberculous Patients' Granulomas Constitute a Nutrient-Rich Reservoir for M. tuberculosis Persistence
Source: PLoS Pathog. 2008 Nov 11;4(11):e1000204. doi: 10.1371/journal.ppat.1000204 (PMC2575403; doi:10.1371/journal.ppat.1000204)

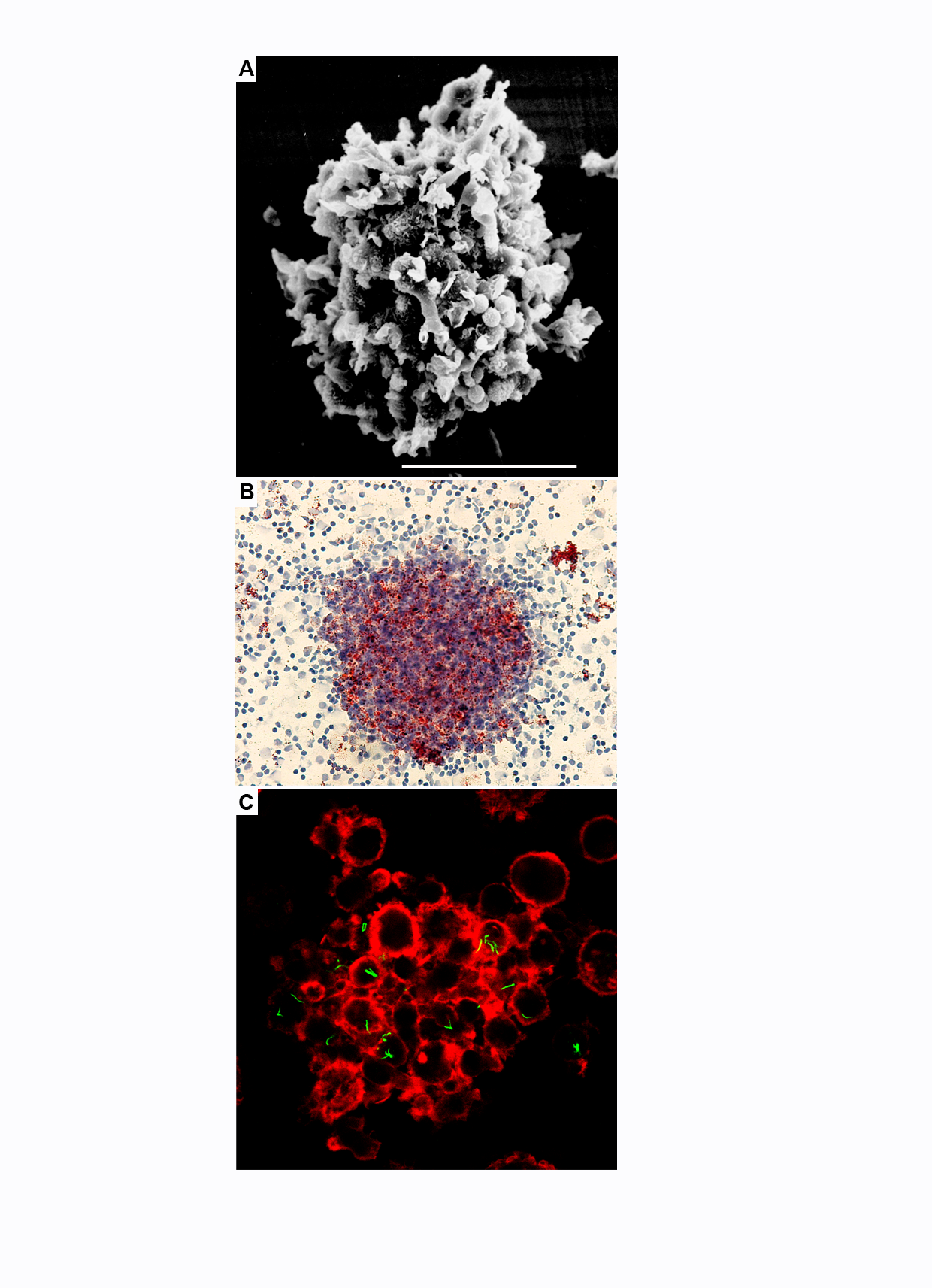

Supplement: Figure S1 — In vitro-induced M.tb granulomas strongly mimic in vivo granulomas. A. Scanning electron microscope observation of a M.tb granuloma 9 days post-infection. The structure of the granuloma is well-defined and -circumscribed, and both macrophages and lymphocytes can be observed around the structure, as is typical of a human granuloma. Bar: 50 µM. B. Peripheral lymphocytes were removed from day 11 granulomas by smooth pipeting, and the remaining structure was stained with Oil red-O and haematoxylin. As in the case of lesion biopsies, a large number of FMs (red staining) can be observed around the central area of in vitro granulomas. Original magnification ×100. C. Confocal microscopy analysis of granulomas induced by a GFP-expressing M.tb strain. To outline the cell contour, the cells were permeabilized and labelled with β-Phaloïdin that stains cortical actin filaments. Original magnification ×400. (2.93 MB TIF) [file ppat.1000204.s001.tif]

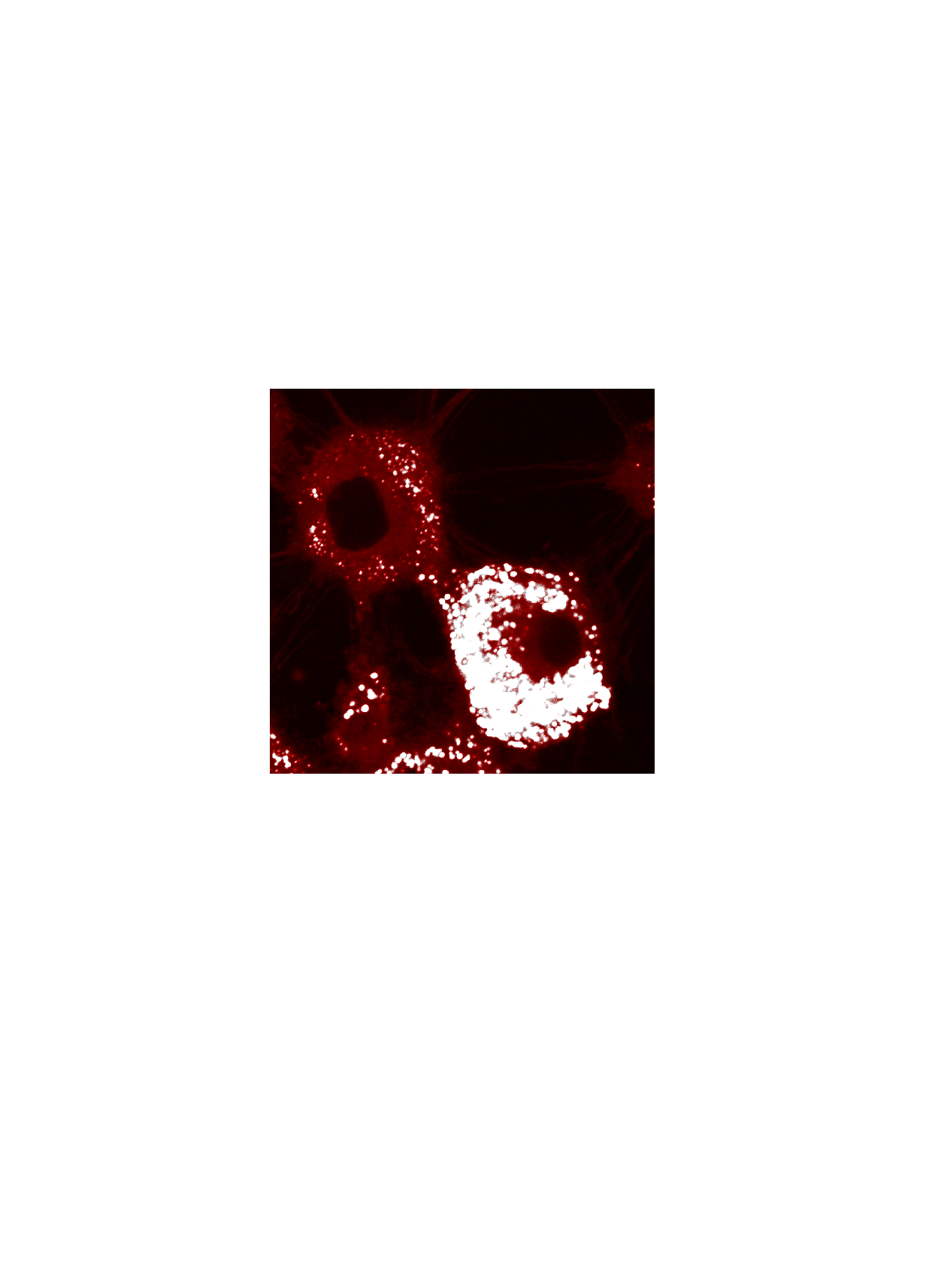

Supplement: Figure S2 — Nile red staining. In order to distinguish the lipids contained within lipid bodies from those of the cell membranes, we took advantage of the fluorescent emission spectrum properties of Nile red which depend upon the lipid Nile red is associated with, i.e. for triacylglycerol: λmax em = 590 nm, for phospholipids: λmax em = 640 nm (Molecular Probes handbook). On confocal microscopy pictures, the phospholipid background of both macrophages and FMs appears in red and the triacylglycerol-rich lipid bodies of FMs appear in white. Cells were considered to be Nile red-positive when more than 50% of the cell surface was stained. Using this criterion, the bottom right cell is a FM and the cell in the upper left corner a macrophage. (1.06 MB TIF) [file ppat.1000204.s002.tif]
